# Clinical Impact of Epithelial-to-Mesenchymal Transition Regulating MicroRNAs in Pancreatic Ductal Adenocarcinoma

**DOI:** 10.3390/cancers10090328

**Published:** 2018-09-13

**Authors:** Sameer Abdallah Dhayat, Max Michael Traeger, Jan Rehkaemper, Anda Jana Stroese, Konrad Steinestel, Eva Wardelmann, Iyad Kabar, Norbert Senninger

**Affiliations:** 1 Department of General, Visceral and Transplantation Surgery, University Hospital Muenster, 48149 Muenster, Germany; m_trae01@uni-muenster.de (M.M.T.); andajana.stroese@ukmuenster.de (A.J.S.); senning@ukmuenster.de (N.S.); 2 Gerhard-Domagk-Institute of Pathology, University Hospital Muenster, 48149 Muenster, Germany; jan.rehkaemper@ukmuenster.de (J.R.); konrad@steinestel.com (K.S.); eva.wardelmann@ukmuenster.de (E.W.); 3 Department of Medicine B, Gastroenterology and Hepatology, University Hospital Muenster, 48149 Muenster, Germany; iyad.kabar@ukmuenster.de

**Keywords:** pancreatic ductal adenocarcinoma, epithelial-to-mesenchymal transition, microRNA, biomarker, epigenetics

## Abstract

Pancreatic ductal adenocarcinoma (PDAC) is one of the most aggressive carcinoma entities worldwide with early and rapid dissemination. Recently, we discussed the role of microRNAs as epigenetic regulators of Epithelial-to-Mesenchymal Transition (EMT) in PDAC. In this study, we investigated their value as diagnostic and prognostic markers in tissue and blood samples of 185 patients including PDAC, non-malignant pancreatic disorders, and age-matched healthy controls. Expression of the microRNA-200-family (microRNAs -141, -200a, -200b, -200c, -429) and microRNA-148a was significantly downregulated in tissue of PDAC Union internationale contre le cancer (UICC) Stage II. Correspondingly, stromal PDAC tissue showed strong expression of Fibronectin, Vimentin, and ZEB-1 (Zinc finger E-box-binding homeobox) versus low expression of E-cadherin. Transient transfection of microRNA-200b and microRNA-200c mimics resulted in the downregulation of their key target ZEB-1. Inversely, blood serum analyses of patients with PDAC UICC Stages II, III, and IV showed a significant over-expression of microRNA-200-family members, microRNA-148a, microRNA-10b, and microRNA-34a. Correspondingly, Enzyme-linked Immunosorbent Assay (ELISA) analyses revealed a significant over-expression of soluble E-cadherin in serum samples of PDAC patients versus healthy controls. The best diagnostic accuracy to distinguish between PDAC and non-PDAC in this patient collective could be achieved in tissue by microRNA-148a with an area under the receiver-operating-characteristic (ROC) curve (AUC) of 0.885 and in blood serum by a panel of microRNA-141, -200b, -200c, and CA.19-9 with an AUC of 0.890. Both diagnostic tools outreach the diagnostic performance of the currently most common diagnostic biomarker CA.19-9 (AUC of 0.834). Kaplan Meier survival analysis of this patient collective revealed an improved overall survival in PDAC patients with high expression of tissue-related microRNA-34a, -141, -200b, -200c, and -429. In conclusion, EMT-regulating microRNAs have great potential as liquid and solid biopsy markers in PDAC patients. Their prognostic and therapeutic benefits remain important tasks for future studies.

## 1. Introduction

Pancreatic ductal adenocarcinoma (PDAC) remains one of the most lethal tumors worldwide with locoregional spread and distant metastasis in about 80%. Rapid disease progression and early metastasis lead to late diagnosis at advanced unresectable stages with an overall cumulative 5-year survival rate below 1% [1,2]. Accumulating evidence has revealed that Epithelial-to-Mesenchymal Transition (EMT) plays a crucial role in the invasion and metastasis of diverse carcinomas, including PDAC [3]. EMT is characterized by a loss of epithelial cell-to-cell contacts and by epithelial cells acquiring motile mesenchymal features leading to cell migration and invasion. At the site of metastases the disseminated mesenchymal tumor cells undergo the reverse transition as Mesenchymal-to-Epithelial Transition (MET) [4,5,6,7]. Metastasis is a complex multistep biological process that is controlled by distinct genes and signaling pathways including EMT-promoting TGF-β and Wnt/β-Catenin pathways [8]. In the case of PDAC, metastasis is one of the main reasons for its fatal prognosis and therefore urgently important in the scientific and clinical work with PDAC. Recently, we reviewed the epigenetic factors that participate in the formation of PDAC metastasis by EMT regulation [9]. Small noncoding microRNAs that modulate gene expression post-transcriptionally have been reported to govern the induction of EMT through regulating its target messenger RNAs (mRNA), e.g., of the E-cadherin transcriptional repressor ZEB-1 (Zinc finger E-box-binding homeobox) [10,11,12,13]. Especially the miR-200-family (miR-141, -200a, -200b, -200c, and -429) [14,15,16], miR-34a [17,18,19], miR-148a [20,21,22,23], miR-203a [24,25,26], and miR-655 [10,27] function strongly EMT-suppressive, whereas miR-10b [28] and miR-197 [29] are shown to promote EMT by targeting mRNAs of crucial proteins of EMT-pathways (Table 3). Furthermore, targeting microRNAs may be a good therapeutic strategy and a promising tool as liquid biopsy markers in PDAC diagnostics and prognosis [30,31]. The multi-target characteristic of microRNAs might be powerful for their use in cancer therapy, because microRNA-based therapy could potentially target many dysregulations in cancer with only one pharmaceutical [32,33]. Gayral et al. summarized the potentials of microRNAs in clinical PDAC management and mentioned possible therapeutical advantages for example in chemosensitization (e.g., miR-141) or in growth and invasion arrest (e.g., miR-34a) [32].

In this clinical study, we focused on the diagnostic and prognostic potential of known EMT-regulating microRNAs in carcinoma tissue and blood serum samples of PDAC patients.

## 2. Results

### 2.1. In-Vitro Expression of EMT-Regulating MicroRNAs and Proteins

**Figure 1:**
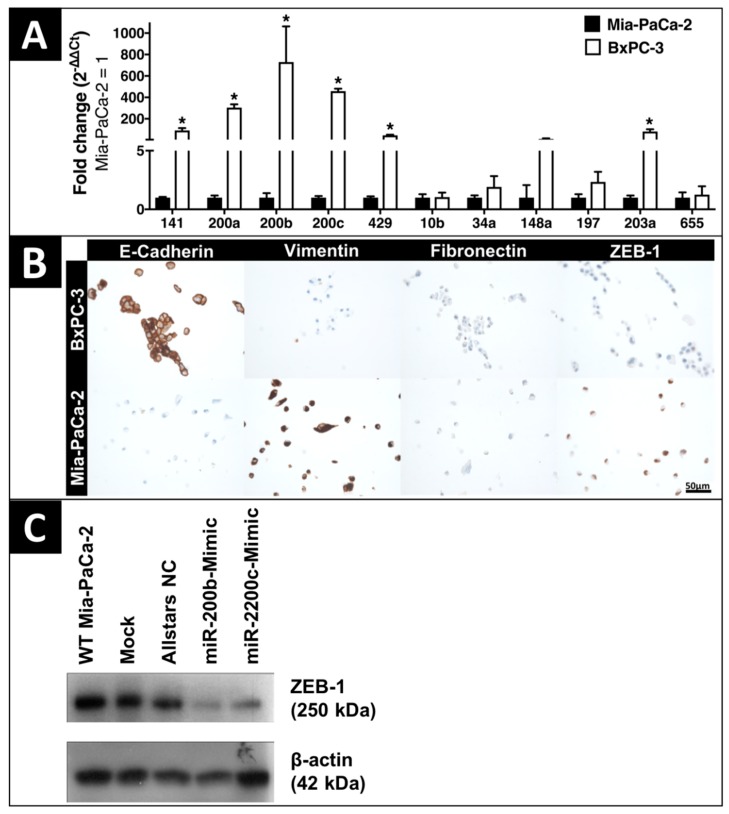
Expression of EMT-regulating microRNAs and EMT-proteins in-vitro. (**A**) EMT-regulating microRNA expression by qRT-PCR in mesenchymal human pancreatic ductal adenocarcinoma (PDAC) cell line Mia-PaCa-2 and epithelial BxPC-3. Fold change values interpreted with the ΔΔCt-method and data expressed as mean ± Standard error of the mean (SEM) (*n* = 3). * indicates significance (*p* ≤ 0.05) against Mia-PaCa-2. (**B**) Immunohistochemistry (IHC) staining of E-Cadherin, Vimentin, Fibronectin and ZEB-1 (Zinc finger E-box-binding homeobox) in human PDAC cell lines Mia-PaCa-2 and BxPC-3. Scale bar: 50 μm. (**C**) EMT-marker protein expression of ZEB-1 by Western blot in mesenchymal wild type (WT) Mia-PaCa-2 after transient transfection with microRNA-200b and -200c mimics.

Phenotype analysis of five certified human PDAC cell lines identified Mia-PaCa-2 with representative mesenchymal spindle-shaped cell morphology and BxPC-3 as characteristic epithelial cell line with plump rounded morphology and enhanced formation of tight cell layers (Figure S1). The expression of microRNA-141 (*p* = 0.002), microRNA-200a (*p* < 0.001), microRNA-200b (*p* = 0.001), microRNA-200c (*p* < 0.001), microRNA-429 (*p* < 0.001), and microRNA-203a (*p* = 0.002) was significantly downregulated in the mesenchymal PDAC cell line Mia-PaCa-2 versus the epithelial cell line BxPC-3 (Figure 1A). The expression of microRNAs -10b, -34a, -148a, -197, and -655 did not show a significant difference between Mia-PaCa-2 and BxPC-3. Correspondingly to their phenotype, immunohistochemistry demonstrated an elevated expression of the mesenchymal marker proteins vimentin, fibronectin, and ZEB-1 in Mia-PaCa-2, whereas epithelial BxPC-3 cells strongly expressed E-cadherin (Figure 1B). Exogenously upregulated microRNA-200b and microRNA-200c by transient transfection with mimics resulted in the downregulation of their key target protein, the transcription factor ZEB-1, in the mesenchymal PDAC cell line Mia-PaCa-2 (Figure 1C).

### 2.2. Clinicopathologic Characteristics

A total of 185 patients including 96 PDAC patients were analyzed (Table S1). There was no difference in age (*p* = 0.474), gender (*p* = 0.133), body mass index (*p* = 0.235), smoking (*p* = 0.333) or alcohol consumption (*p* = 0.233) between PDAC and its control groups of chronic pancreatitis, intraductal papillary mucinous neoplasm (IPMN), and healthy volunteers. Diabetes mellitus (*p* = 0.024) and elevated CA.19-9 (*p* < 0.001) correlated significantly with PDAC. Sub-group analysis of PDAC patients revealed an improved overall survival for low grading (*p* = 0.006), no synchronous metastasis (*p* < 0.001), curative surgery (*p* < 0.001), UICC Stage II with a median overall survival of 26.9 months versus 5.1 months for UICC Stage IV (*p* < 0.001), and adjuvant chemotherapy (*p* < 0.001) (Table 1). The median post-surgical follow-up of PDAC patients was 17 months.

### 2.3. Expression of EMT-Regulating MicroRNAs in Clinical Solid and Liquid Biopsies

**Figure 2:**
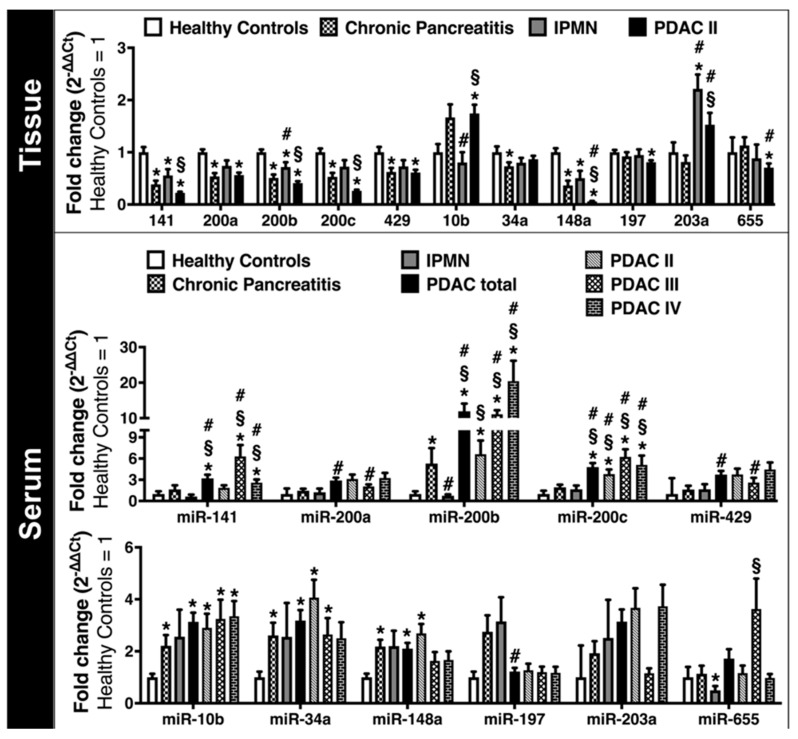
EMT-regulating microRNAs in human pancreatic tissue and blood serum samples. Fold change values for tissue and blood serum samples analyzed with qRT-PCR and interpreted with the ΔΔCt-method. Data expressed as the mean ± SEM. * indicates significance (*p* ≤ 0.05) against healthy controls; **#** against chronic pancreatitis and **§** against intraductal papillary mucinous neoplasm (IPMN).

Macrodissected tissue of PDAC UICC Stage II revealed a significant down-regulation of microRNA-141 (*p* < 0.001), microRNA-200a (*p* = 0.005), microRNA-200b (*p* < 0.001), microRNA-200c (*p* < 0.001), microRNA-429 (*p* = 0.017), microRNA-148a (*p* < 0.001), microRNA-197 (*p* = 0.001), and microRNA-655 (*p* = 0.019) versus healthy controls (Figure 2). Additionally, microRNA-148a, microRNA-203a and microRNA-655 could significantly (*p* = 0.002; *p* = 0.028; *p* = 0.052) discriminate between PDAC and chronic pancreatitis. MicroRNA-141 (*p* = 0.022), microRNA-200b (*p* = 0.001), microRNA-200c (*p* = 0.003), and microRNA-148a (*p* < 0.001) were significantly down-regulated in PDAC versus IPMN. In PDAC tissue microRNA-10b was up-regulated versus IPMN (*p* = 0.007) and healthy controls (*p* = 0.019). MicroRNA-34a could discriminate between healthy controls and chronic pancreatitis (*p* = 0.011), but not versus PDAC. Notably, microRNA-148a could significantly discriminate both PDAC patients and healthy controls from all other study groups.

Inversely to the microRNA expression in PDAC tissues, blood serum analysis of patients with PDAC UICC Stage II, III, and IV (as single sub-groups and all together as “PDAC total”) showed a significant over-expression of microRNA-141 (*p* = 0.038), microRNA-200b (*p* < 0.001), microRNA-200c (*p* < 0.001), microRNA-10b (*p* < 0.001), microRNA-34a (*p* = 0.001), and microRNA-148a (*p* = 0.017) compared to healthy controls. Subgroup analysis of the different PDAC UICC Stages revealed significant upregulation of microRNA-10b (*p* = 0.001), microRNA-34a (*p* < 0.001), microRNA-148a (*p* = 0.003), microRNA-200b (*p* = 0.001), and microRNA-200c (*p* = 0.001) in UICC Stage II; of microRNA-10b (*p* = 0.002), microRNA-34a (*p* = 0.045), microRNA-141 (*p* = 0.013), microRNA-200b (*p* < 0.001), and microRNA-200c (*p* < 0.001) in UICC Stage III; and of microRNA-10b (*p* = 0.002), microRNA-141 (*p* = 0.022), microRNA-200b (*p* < 0.001), and microRNA-200c (*p* = 0.004) in UICC Stage IV compared to healthy controls. Additionally, circulating microRNA-141 (*p* = 0.041), microRNA-200a (*p* = 0.039), microRNA-200b (*p* = 0.018), microRNA-200c (*p* = 0.001), microRNA-429 (*p* = 0.027), and microRNA-197 (*p* = 0.040) could significantly discriminate between PDAC and chronic pancreatitis (Figure 2). Correlation analysis between status of metastasis and circulating EMT-regulating microRNAs in patients with PDAC UICC stages II, III, and IV did not show any significant correlation (*p* > 0.05 in Spearman).

### 2.4. Expression of EMT-Marker Proteins in Human Pancreatic Tissue and Blood Serum Samples

**Figure 3:**
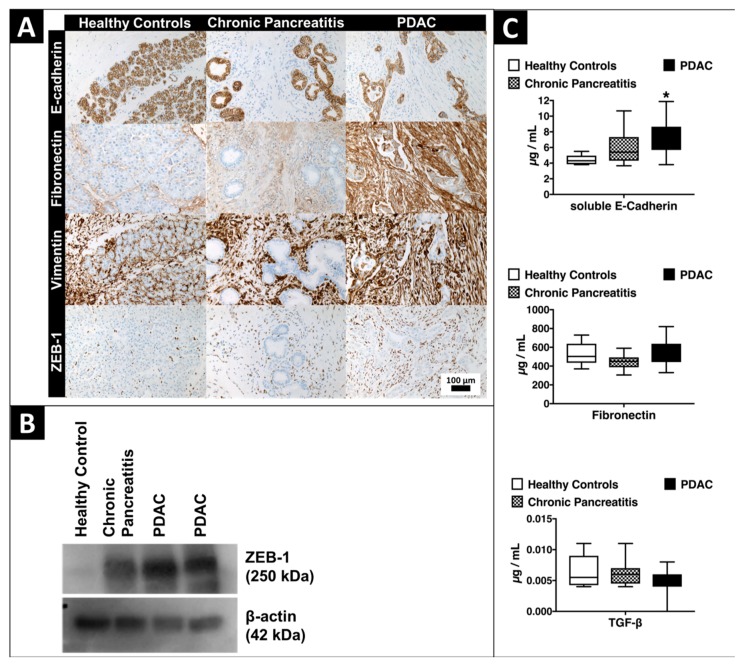
EMT-marker protein expression in human pancreatic tissue and blood serum samples. (**A**) IHC staining of the epithelial marker E-cadherin, mesenchymal markers vimentin, fibronectin and the transcription factor ZEB-1 in human PDAC and healthy pancreatic tissue. Scale bar: 100μm. (**B**) Protein expression of ZEB1 in macrodissected tissue specimens analyzed with Western blot. (**C**) EMT-marker protein analyzed in blood serum samples by Enzyme-linked Immunosorbent Assay (ELISA). * indicates significance (*p* ≤ 0.05) against healthy controls.

Correspondingly to our findings of EMT-marker expression in vitro, stromal PDAC tissue showed strong expression of Fibronectin, Vimentin and ZEB-1 versus low expression of E-cadherin. Immunohistochemistry proofs E-cadherin to be an epithelial marker that is expressed in the cytoplasm of epithelial pancreatic ductal cells and shows that vimentin antithetically is strongly expressed in the cytoplasm of surrounding stromal tissue cells in tissue samples over all entities. Fibronectin is expressed in the cytoplasm of epithelial and obviously stronger in the cytoplasm of the surrounding stromal cells. The transcriptional regulator ZEB-1 is expressed in the nuclei of stromal cells (Figure 3A). Western blot analysis of ZEB-1 as the key target protein of the microRNA-200 family showed a strong over-expression in macrodissected cryopreserved PDAC tissue versus healthy pancreatic tissue (Figure 3B). Enzyme-linked Immunosorbent Assay (ELISA) analyses of soluble E-Cadherin, fibronectin and TGF-beta revealed a significant over-expression of soluble E-Cadherin (*p* < 0.001) in serum samples of patients with PDAC versus healthy volunteers (Figure 3C).

### 2.5. Diagnostic Impact of EMT-Regulating MicroRNAs

**Figure 4:**
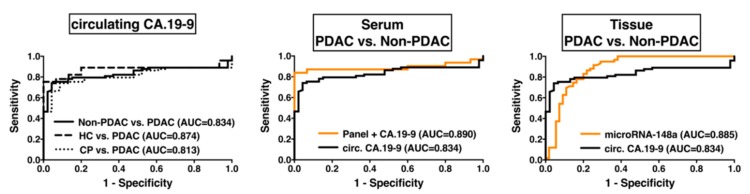
Diagnostic potential of EMT-microRNAs by receiver-operating-characteristic (ROC) analysis in tissue and blood serum samples of PDAC Union internationale contre le cancer (UICC) Stages II-IV.

Diagnostic potential of EMT-regulating microRNAs in blood serum and tissue was analyzed by the receiver-operating-characteristic (ROC) curve analysis (Figure 4). As the point of comparison, we used the diagnostic performance of the currently most-used liquid PDAC biomarker CA.19-9 in blood serum samples of our total patient collective. Circulating CA.19-9 was a good discriminator between PDAC and healthy control (area under the ROC-curve (AUC) = 0.874; *p* < 0.001) and between PDAC and chronic pancreatitis (AUC = 0.813; *p* < 0.001) and could discriminate PDAC from non-PDAC samples (including healthy controls, chronic pancreatitis and IPMN) with good accuracy (AUC = 0.834; *p* < 0.001) presenting a sensitivity of 0.781 and a specificity of 0.870 (Likelihood Ratio: 5.97). The best diagnostic accuracy for single EMT-regulating microRNAs to distinguish between PDAC and healthy pancreatic tissue was shown for microRNA-148a (AUC = 0.996; *p* < 0.001), microRNA-200b (AUC = 0.869; *p* < 0.001), and microRNA-200c (AUC = 0.950; *p* < 0.001) with excellent accuracy.

In blood serum, circulating microRNA-200b (AUC = 0.869; *p* < 0.001), microRNA-200c (AUC = 0.790; *p* < 0.001), and microRNA-34a (AUC = 0.745; *p* = 0.003) could differentiate patients with PDAC and healthy volunteers with good to fair accuracy. The best diagnostic accuracy to distinguish between PDAC and non-PDAC (including healthy controls, chronic pancreatitis, and IPMN) could be achieved in tissue by microRNA-148a with an AUC of 0.885 (*p* < 0.001, sensitivity: 0.831, specificity: 0.800, Likelihood Ratio: 4.153) and in blood serum by the combination of a panel of microRNA-141, -200b, -200c, and CA.19-9 with an AUC of 0.890 (*p* < 0.001, sensitivity: 0.871, specificity: 0.933, Likelihood Ratio: 13.06). The analysis of the diagnostic performance discriminating all non-PDAC samples from PDAC patients is shown in Table 2 for all single EMT-miRs and CA.19-9.

### 2.6. Potential Prognostic Impact of EMT-Regulating MicroRNAs

**Figure 5:**
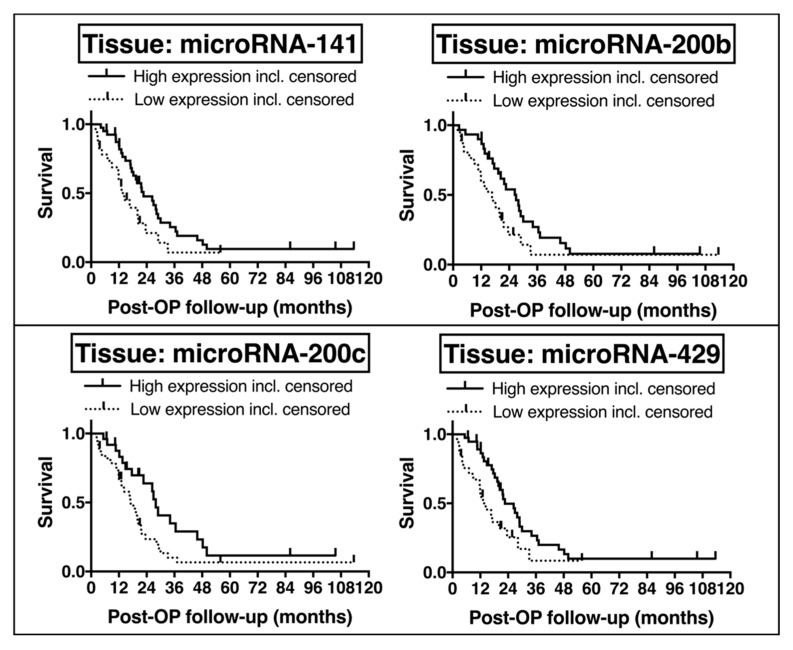
Prognostic impact of EMT-microRNAs by Kaplan-Meier analysis of overall survival in tissue of PDAC UICC Stage II.

Univariate Kaplan-Meier survival analysis revealed a significantly improved overall survival in PDAC patients with high tissue expression of microRNA-34a (median ΔCt cut-off 8.63; *p* = 0.019; median survival: 22.64 months (high) vs. 11.89 months (low)), microRNA-141 (median ΔCt cut-off 9.95; *p* = 0.038; median survival: 22.64 months (high) vs. 14.29 months (low)), microRNA-200b (median ΔCt cut-off 6.30; *p* = 0.036; median survival: 26.94 months (high) vs. 16.66 months (low)), microRNA-200c (median ΔCt cut-off 4.97; *p* = 0.025; median survival: 27.96 months (high) vs. 17.12 months (low)), and microRNA-429 (median ΔCt cut-off 11.93; *p* = 0.025; median survival: 22.64 months (high) vs. 14.29 months (low)) (Figure 5). Subgroup analysis of the groups with low and high tissue-related microRNA expression levels revealed homogenous distribution of number of patients and no significant correlation of subgroups and age, gender, body mass index, pre-surgical diabetes mellitus, tumor size, tumor grade, nodal invasion, resection margin, and adjuvant chemotherapy (*p* > 0.05 by Spearman correlation), except for number of patients and body mass index for tissue microRNA-34a, and nodal invasion for tissue microRNAs-141, -200c and -429.

Analysis of all PDAC serum samples (including PDAC UICC stages II, III, and IV) by univariate Kaplan-Meier survival analysis showed an improved overall survival for high expression of circulating microRNA-34a (median ΔCt cut-off 8.23; *p* = 0.038) and circulating microRNA-655 (median ΔCt cut-off 13.60; *p* = 0.040). However, this analysis may have a bias by the fact that different PDAC stages are included. Subgroup analysis only for PDAC UICC stage II patients did not reveal significant results in the univariate Kaplan-Meier survival analysis. The limited study power did not allow further survival analyses by Cox regression.

## 3. Discussion

A plethora of preclinical studies could demonstrate that EMT is a process closely associated with tumor progression, metastasis, and prognosis in gastrointestinal cancer including PDAC. However, translational applicability of EMT markers into the clinical setting of oncological diseases still remains a controversial issue. There is increasing evidence that microRNAs are not only key regulators in malignant EMT but have also impact as potential biomarkers with high stability to laboratorial processes in PDAC [31,32,34,35,36,37,38]. There is urgent need for PDAC-specific non-invasive biomarkers at early tumor-resectable UICC Stage in order to improve patient prognosis. This is particularly important as the current most-common liquid biomarker CA.19-9 has certain limitations like poor specificity [39].

**Figure 6:**
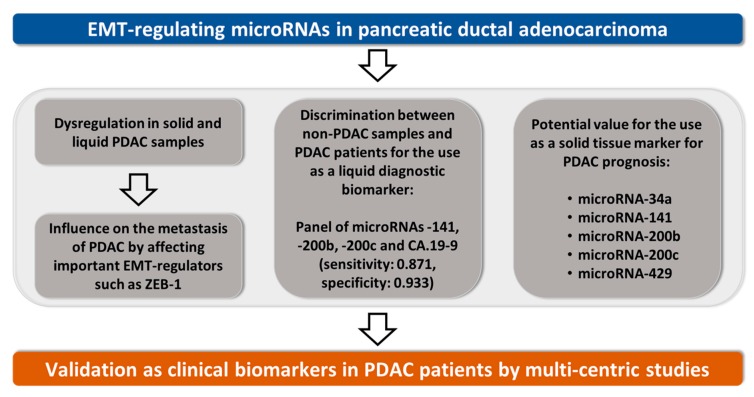
Scheme of the main findings of this study.

In this current study we deeply investigated specific EMT-regulating microRNAs and analyzed their clinical impact on diagnosis and prognosis in PDAC patients (Figure 6). The dysregulation of EMT-regulating microRNAs in tissue and blood serum samples of PDAC patients has been widely shown in the scientific field [9]. However, there is a discrepancy of microRNA expression in PDAC tissue and corresponding preoperative blood serum samples. Our inverse and apparently contradicting expression data of EMT-related microRNAs, particularly the microRNA-200 family, are in accordance with results of previous tumor marker studies [12,40,41,42]. It is supposed that the increase of circulating microRNA-200 is the result of more circulating tumor cells (CTC) with predicted worse survival in various cancers [43,44,45]. Furthermore, Le et al. proposed that microRNA-200 family members are secreted by highly metastatic epithelial cancer cells promoting metastatic progress via extracellular vesicles [46]. Interestingly, it is reported that improved overall survival correlated positively with circulating microRNA-200 expression in patients with ovarian and colorectal carcinomas [47,48]. Therefore, CTC-related or exosomal microRNA-200 might be interesting targets of future studies. Considering, that the microRNA-200 family members function as tumor suppressor genes, their different regulation in PDAC tissue and circulation will remain a subject of further research.

As previously reviewed, the microRNA-200-family is one of the main epigenetic regulators of EMT [16,49]. Our results confirm a strong dysregulation of the microRNA-200-family and their target proteins E-cadherin, vimentin, and fibronectin in PDAC. Furthermore, our in vitro results proclaim the well-known pathway of microRNA-200-family members being strongly involved in the process of EMT by suppressing E-cadherin’s transcription repressor ZEB-1 [14]. Similarly, Bracken et al. showed a reciprocal negative regulation between ZEB-1 and the microRNA-200-family that is capable of inducing an epithelial respectively mesenchymal phenotype by over-expression or inhibition of one part [14,50]. Brabletz et al. explained that the microRNA-200-family also targets molecules in Notch signaling, that mediates tissue homeostasis and is linked to EMT, and showed up-regulation of Notch signaling and ZEB-1 expression with simultaneous down-regulation of microRNA-200-family in PDAC [11]. Highly important for research and clinic, ZEB-1 plays a crucial role in PDAC development and especially metastasis, as shown by Krebs et al. in the murine KPC model organism [13]. Furthermore, ZEB-1 is necessary for PDAC initiation and inhibits other stemness-repressors including microRNA-200-family and microRNA-203 [51] and seems to play a pivotal role in drug resistance [52]. Especially our findings regarding the diagnosis of PDAC at early tumor stages are promising and clinically relevant. A panel of circulating microRNAs consisting of microRNA-141, -200b, and -200c could increase the diagnostic accuracy of CA.19-9 to a sensitivity of 87% and a specificity of 93%. Furthermore, elevated expression of these microRNA-200-family members in PDAC tissue correlated with a significantly improved overall survival in PDAC patients in univariate analysis. However, the statistical power of this monocentric study was unsatisfactory to reach significance in multivariate survival analysis.

Besides the microRNA-200-family, microRNA-148a showed the strongest dysregulation of all tested microRNAs in our tissue collective and by far the best diagnostic accuracy to distinguish between PDAC and non-PDAC with an AUC of 0.885. Xia et al. reviewed a similar expression pattern and promising results for microRNA-148a in gastric cancer [53] as well as Pan et al. in hepatocellular carcinoma [54]. Previous studies on PDAC demonstrated a significant down-regulation of microRNA-148a in PDAC tissue samples as well and shed light on the connection of microRNA-148a and PDAC invasion, progression, and metastasis [20,55,56]. The high potential of microRNA-148a as a clinical marker for diagnosis and prognosis was also shown in other carcinomas such as osteosarcoma [57], non-small cell lung cancer [58], and laryngeal carcinoma [59]. MicroRNA-34a seems to be a promising candidate for a prognostic biomarker in PDAC as well. In our patient collective, high miR-34a expression in tissue correlated with significantly improved overall survival. These results are in accordance with publications showing a better overall survival for high microRNA-34a expression in different types of solid cancers including PDAC [60,61,62]. A recent study by Tang et al. illuminated its influence on the process of EMT in PDAC by post-transcriptional regulation of the EMT-regulators SNAIL1 and Notch1 [17] while Ahn et al. reveal microRNA-34a as a target of ZEB-1 and showed its tumor-suppressive functions by decreasing tumor cell invasion and metastasis [18]. Ji et al. demonstrated a significant reduction of tumor initiating cells and inhibition of tumor growth in vitro and in vivo after restoration of physiological microRNA-34a expression [63]. Moreover, microRNA-34a shows very high potential for being part in clinical cancer therapy and is part of pre-clinical and clinical studies evaluating the therapeutical possibilities in different types of cancer including PDAC [64]. Correspondingly to studies by Harazono et al. in the PDAC cell line Panc-1 and in esophageal squamous cell carcinoma tissue highlighting ZEB-1 as a target of microRNA-655, we identified circulating microRNA-655 as an EMT-suppressive microRNA correlating significantly with improved overall survival in PDAC patients [27]. Besides the tested EMT-regulating microRNAs, other microRNAs have been connected with great potential as biomarkers in PDAC as well. Among others, microRNAs -21, -155, -196a and -210 are shown to be potential non-invasive biomarkers for diagnosis, prognosis and/or therapy response [19,65,66,67,68,69,70,71,72,73]. Moreover, microRNA-21 antisense oligonucleotides are also linked with high potential in PDAC therapy as a partner for gemcitabine [74].

Taken together, the results in our patient collective suggest the potential of the EMT-suppressors microRNA-34a, -141, -148a, -200b, -200c, and -655 targeting ZEB-1 as clinical biomarkers for PDAC. Other studies showed similar potential in PDAC especially for microRNA-34a [19,60] and microRNA-200-family members [75,76]. Further studies on the potential of EMT-regulating microRNAs as tools in PDAC therapy are on-going. The delivery of therapeutics containing microRNAs and/or anti-miRs is a complex issue, but approaches for combinations with nanoparticles showed promising potential to overcome this problem [30,77]. Of the tested EMT-regulating microRNAs, especially miR-34a might be useful in PDAC therapy by attenuating tumor growth [63,78]. Using microRNA-34a and a lipid-based nanoparticle delivery system resulted in significant tumor growth inhibition both in Mia-PaCa-2 subcutaneous xenografts and in an orthotopic pancreatic setting [79]. Trang et al. showed a 60% reduction of tumor area in mouse models of non-small cell lung cancer after treatment with microRNA-34a mimics delivered with neutral lipid emulsion via tail vein injection [80]. Correspondingly, microRNA-34a based therapy using intravenous injection of T-VISA-miR-34a:liposomal complex nanoparticles resulted in inhibition of tumor growth and better overall survival without systemic toxicity in murine breast cancer models [81]. As microRNAs showed their ability in chemosensitization it seems to be another auspicious path to use them in combination with chemotherapy [82,83,84]. A main challenge but also dramatic potential for the clinical use of microRNAs in cancer therapy lies in the fact that one single microRNA can target multiple mRNAs effecting several crucial biological processes.

So far, the evidence for EMT and its role in PDAC metastasis [85] led us to consider that the development of EMT inhibitors might provide opportunities for PDAC treatment.

## 4. Materials and Methods 

### 4.1. Patients and Samples

A biobank of tissue and blood samples combined with a clinical follow-up database was maintained prospectively by the Department of General, Visceral and Transplantation Surgery and the Comprehensive Cancer Center Muenster of the University Hospital Muenster, Germany. Between 2003 and 2017, tissue specimens and blood serum samples of 185 patients with PDAC UICC Stage II (65× tissue, 22× serum); PDAC UICC Stage III (11× serum); PDAC UICC Stage IV (16× serum); intraductal papillary mucinous neoplasm (IPMN, 9× tissue, 6× serum); chronic pancreatitis (21× tissue; 16× serum); benign, non-inflammatory pancreatic specimens (29× tissue); and age-matched healthy controls (17× serum) were collected (see Table S1). The study group of benign, non-inflammatory pancreatic specimens consisted of 29 tissue samples of patients with cystadenoma of the pancreas, that have been taken during surgery in fair distance to the cystadenoma as they function as the healthy control group in the tissue analysis. Of some patients both tissue and blood serum were sampled (*n* = 27). Patients that received immunosuppression, chemo- or radiotherapy before blood sampling and/or surgery were excluded to avoid potential influences on microRNA expression. Patients with a second tumor entity or pancreatic neuroendocrine tumor were excluded as well. Collection, processing and storage of venous blood samples and intraoperatively obtained tissue samples were performed under standardized conditions as described previously [34,86,87]. All tissue specimens showed >60% viable cells and <20% necrosis with high percentage of cancer cells in guided macrodissection of PDAC samples proofed by two experienced pathologists. Ethical approval for tissue and/or blood serum collection was obtained by the Ethics committee of the University Muenster (1IXHai, 11.8.2011) and all patients provided informed written consent. All patients with suspicion of resectable PDAC underwent radical resection and were assigned to pancreatic head resection, total pancreatectomy, pancreatic left resection or excisional tumor biopsy followed by approved adjuvant therapy. Clinical data, histopathological information and survival follow-up data were collected for all patients.

### 4.2. Selection of MicroRNAs and Their Target Proteins

On the basis of our previous review on epigenetic regulation by EMT-regulating microRNAs in PDAC and further literature research we analyzed the following microRNAs in solid and liquid biopsies (Table 3).

Then we focused on the well-known EMT markers ZEB-1, E-Cadherin, soluble E-Cadherin, vimentin and fibronectin. MicroRNA expression data of tissue specimens were normalized to expression levels of the three housekeeping genes RNU1A, SNORD68, and SNORD96A, selected from a total of 10 tested housekeeping genes in PDAC tissue (Figure S2) analyzed by geNorm Software (Biogazelle NV, Zwijnaarde, Belgium). GeNorm calculates the average gene expression stability measure M for a reference gene by stepwise exclusion of the most unstable reference gene. In the ranking of reference genes, housekeeping genes with an average gene expression stability ≤ 0.5 have a high expression stability. Circulating microRNA expression data were normalized to the synthetic microRNA-39 from Caenorhabditis elegans (cel-microRNA-39) as a spike-in control.

### 4.3. RNA Isolation and Quantification of EMT-Regulating MicroRNAs

Before PDAC and Non-PDAC tissue samples have been used for further analysis, a representative hematoxylin and eosin (H&E)-stained section for each histological sample was reviewed by a trained and experienced pathologist. Tissue samples included in this study had >60% viable cells and <20% necrosis. Tumor and stromal tissue areas were selectively distinguished with a permanent marker by the pathologist to guide macrodissection with high tumor cell proportion in PDAC samples. Macrodissection of samples with adenoma was carried out to create benign controls using sample material distant from adenoma. Tumor macrodissection with RNA purification from each formalin-fixed paraffin-embedded (FFPE) tissue sample through robotic workstation (QIAcube, Qiagen, Hilden, Germany) and total RNA isolation from cryopreserved blood serum samples using QIAzol Lysis Reagent (Qiagen) as a part of the miRNeasy Serum/Plasma Kit (Qiagen) was realized as described previously [34,86]. Quantitative Real-Time (qRT) PCR was performed using the miScript PCR system (Qiagen) as described previously [34,86,87]. Quantitative microRNA analysis was performed using CFX Manager Software v2.1 (Bio-Rad Laboratories, Munich, Germany). Expression of microRNA-10b, -34a, -141, -148a, -197, -200a, -200b, -200c, -203a, -429, and -655 was analyzed quantitatively relative to the housekeeping genes by the ΔΔCt (cycle threshold) method [88].

### 4.4. Tissue Array Immunohistochemistry

Immunohistochemical staining of 3 µm thick macrodissected and tissue-arrayed formalin-fixed paraffin embedded (FFPE) tissue sections was performed automatically using the Benchmark Ultra (Ventana Medical Systems, Inc., Oro Valley, AZ, USA). This staining machine contains peroxidase, inhibitors, buffer solutions, dye and the secondary antibody (OptiView HQ Universal Linker, Ventana Medical Systems). E-cadherin, vimentin (each monoclonal, ready-to-use, Ventana Medical Systems), fibronectin (polyclonal, 1:1000, Agilent Technologies, Santa Clara, CA, USA) and ZEB-1 (polyclonal, 1:400, Sigma-Aldrich, St. Louis, MO, USA) were used as primary antibodies. Negative controls without primary antibodies and positive controls (pancreas, tonsil, colon and kidney) were included in all experiments using the same experimental conditions. The slides were counterstained with Haematoxylin and dehydrated in graded alcohols. Immunohistochemical staining was evaluated separately in stromal and epithelial tissue by two pathologists in a blinded manner using light microscopy (BX51, Olympus) [89].

### 4.5. Cell Lines and MicroRNA Transfection

The five certified human PDAC cell lines Mia-PaCa-2, Panc-1, BxPC-3, SU.86.86, and AsPC-1 were purchased from the American Type Culture Collection (ATCC; Rockville, MD, USA) and were cultured as described previously [72]. MicroRNA expression was analyzed by qRT-PCR as described for clinical tissue samples. After analysis of the phenotypes and EMT-microRNA expression profiles in these five PDAC cell lines, the most epithelial cell line BxPC-3 and the most mesenchymal cell line Mia-PaCa-2 have been used for further analysis. EMT-marker protein expression was analyzed by immunohistochemistry using the same antibodies as for tissue. The cells were fixed in 4% Formalin and added to a PBS/Agarose mix (0.25 g Agarose in 25 mL of PBS), and then processed into FFPE-blocks. Evaluation of staining was conducted in a blinded manner using light microscopy (Eclipse E1000M and NIS-Elements D3.1 Imaging software, Nikon, Tokyo, Japan) as described previously [34]. Mia-PaCa-2 as the PDAC cell line with the lowest microRNA-200b and -200c expression was transiently transfected with miScript miRNA-200b and -200c mimics (Qiagen, Hilden, Germany) using HiPerFect transfection reagent (Qiagen, Hilden Germany). As recommended by the manufacturer, 4 × 10^5^ cells were seeded in appropriate growth medium per well of a 6-well plate. A transfection complex consisting of HiPerFect, Opti-MEMTM I Reduced Serum Medium (Gibco, Carslbad, CA, USA) and microRNA mimic was added to the cells at a final concentration of 5 nM of mimic. As negative transfection control miScript Allstars NC or Inhibitor NC (Qiagen, Hilden, Germany) were transfected instead. Cells were cultivated at standard conditions for 48 h.

### 4.6. Western Blotting

Cryopreserved tissue samples and total cell lysates were dissociated 48 h post transfection in RIPA lysis buffer with 1× Protease Inhibitor Cocktail (Cell Signaling Technology, Cambridge, UK) using a TissueLyserLT bead mill (Qiagen, Hilden, Germany). The lysates were cleared by centrifugation at 14,000 *g* for 45 min at 4 °C. After quantification using Pierce^TM^ BCA Protein Assay Kit (ThermoScientific^TM^, Waltham, MA, USA), proteins were separated by sodium dodecyl sulfate (SDS)—6% polyacrylamide gel electrophoresis and blotted to a blocked PVDF membrane (Merck, Darmstadt, Germany). The primary antibody anti-ZEB1 (anti-rabbit, 1:500, HPA027524, Sigma-Aldrich, St. Louis, MO, USA) was diluted in blocking buffer and incubated overnight at 4 °C. Rabbit anti-actin (A2066, Sigma-Aldrich, St. Louis, MO, USA) was used as a loading control at 1:2000. The membrane was incubated with secondary antibody IgG-HRP (anti-rabbit, 1:14,000, A6154, Sigma-Aldrich) for 1 h at room temperature. After three washings, peroxidase was detected using ImmobilonTM ECL western blotting substrate (Millipore, Schwalbach, Germany).

### 4.7. Enzyme-Linked Immunosorbent Assay (ELISA)

Quantification of EMT-marker proteins in representative human blood serum samples of patients with PDAC (*n* = 16), chronic pancreatitis (*n* = 8) and age-matched healthy controls (*n* = 8) was conducted with ELISA for soluble E-cadherin (ready-to-use kit 99-1700, 1:6.67, Invitrogen, Carlsbad, CA, USA), fibronectin (ready-to-use kit EHFN1, 1:10,000, ThermoScientific, Waltham, MA, USA) and TGF beta 1 (ready-to-use kit ab100647, abcam, Cambridge, UK) as written in the fabricants’ manuals.

### 4.8. Statistical Analysis

Explorative and descriptive statistical analysis of patients’ data, microRNA expression, survival analysis, and diagnostic potential were performed with Microsoft^®^ Excel for Mac Version 16 (Microsoft Corp., Redmond, WA, USA), SPSS^®^ Statistics Version 24 (IBM Corp. Armonk, NY, USA) and GraphPad Prism 7 (GraphPad Software, INC, La Jolla, CA, USA). At study initiation power estimation determined a sample size of at least 15 samples per study group. Post hoc power analysis by G*Power 3.0 recommended the inclusion of at least 159 patients with 53 patients per each of the three study groups of PDAC, healthy and chronic pancreatitis to achieve a power 1-β of 80% [90].

Patients’ dataset was analyzed with unpaired two-tailed t-test and PDAC patients’ survival analysis regarding histopathologic characteristics with Log-rank (Mantel-Cox). For microRNA expression analysis outliers within one group of more than two standard deviations have been excluded for graph generation and analysis of significance that has been done by the comparison of two groups using unpaired two-tailed t-test. Correlation analysis between circulating microRNA-expression and metastasis was conducted using Spearman test. Patients were categorized into low microRNA- and high microRNA-categories at individually set cut-off ΔCt-values based on the median. The diagnostic potential of microRNAs was analyzed by receiver-operating-characteristic (ROC) method. Box-and-whisker plots demonstrating the median (middle quartile) were used to show the ELISA results. Overall survival and relapse-free survival were the primary end points, as measured from the date of surgery to the time of cancer-related death or tumor relapse or the last routine follow-up examination by the Department of General, Visceral and Transplantation Surgery respectively. Data of patients who were still alive and without evidence of tumor relapse at the end of the study were censored. Survival data was tested for significance using Log-rank (Mantel-Cox) test and plotted for Kaplan-Meier curves. A Cox proportional-hazards regression model was used to estimate hazard ratios and 95% confidence intervals (CIs) and to perform multivariate survival analysis using a forward stepwise variable selection procedure based on the likelihood ratio. Variables with significant *p*-values in the univariate analysis were included in the multivariate analysis. Values for *p* ≤ 0.05 were considered to be statistically significant for this patient collective.

### 4.9. Limitations of This Study

In the context of reporting recommendations for tumor marker prognostic studies (REMARK) by McShane et al. [91] relevant information is provided about the study design, preplanned hypotheses, patient and specimen characteristics including control samples with inclusion and exclusion criteria, assay methods with references of detailed protocols and quality controls in this study. Methods of statistical analysis together with details to distributions of demographic characteristics, disease-specific prognostic variables, and EMT-related microRNAs, as well as overall and subgroup numbers of patients and of events are reported. However, the statistical power of this monocentric study with retrospective data analysis was unsatisfactory for Non-PDAC control groups in tissue and serum analyses and for each single PDAC UICC Stage in serum analysis to determine the prognostic impact of the 11 EMT-related microRNAs in PDAC tissue and serum samples. Furthermore, no valid statement about the prognostic impact of immunohistochemically examined EMT proteins and their correlation with corresponding microRNA expression could be made. The used method of tissue array immunohistochemistry only offers qualitative information on the incongruence of epithelial and mesenchymal protein expression in each study group with equally distributed epithelial and stromal proportions, but lacks quantifiable details. A correlation of the malign and benign study groups with highly different proportions of epithelial and stromal proportions was not indicated. However, we could validate and confirm the well-known high expression of mesenchymal proteins in PDAC tissue and of epithelial proteins in healthy pancreatic tissue. Overall, we could demonstrate the high potential of specific EMT-related microRNAs in PDAC tissue and blood serum as diagnostic markers. Further multi-center studies with a larger number of patients are required to reevaluate the impact of microRNA-200 family members, microRNA-34a, and microRNA-148a as diagnostic and survival biomarkers in PDAC.

## 5. Conclusions

This study indicates the high potential of EMT-related microRNAs as solid and liquid biomarkers for PDAC. Our results strongly underline the importance of microRNA-dysregulation in PDAC carcinogenesis and its high potential for clinical use. Especially the members of the microRNA-200-family and microRNA-148a seem to be promising candidates for translational use in the management of patients with pancreatic disorders and urgently call for further multi-center studies.

## Figures and Tables

**Table 1:** Histopathologic characteristics of PDAC patients. Indication of median overall survival in months and 95% confidence interval (CI). *p*-values are calculated by Log-rank (Mantel-Cox). *p* ≤ 0.05 indicates significance.

Category	Number of PDAC Patients	Median Overall Survival (Months)	95% CI	*p*-Value
**Total**	96	17.1	12.6–21.6	
**Age (years)**				0.097
≤60	35	21.8	9.5–34.2	
>60	61	14.9	9.9–19.9	
**Gender**				0.594
Female	57	16.5	11.7–21.3	
Male	39	18.3	12.621.6	
**Body Mass Index**				0.932
≤25	57	16.5	12.0–21.0	
>25	37	18.3	11.4–25.1	
**Smoker**				0.245
Yes	22	11.8	6.1–17.4	
No	74	18.3	14.7–21.8	
**Alcohol abusus**				0.100
Yes	5			
No	91	17	13.0–20.9	
**Pre-surgical Diabetes mellitus**				0.865
Yes	31	14.9	7.1–22.4	
No	65	17.1	14.0–20.3	
**Pre-surgical pancreatitis**				0.094
Yes	15	13.1	7.9–14.8	
No	81	18.3	14.7–21.8	
**Pre-surgical CA.19-9 (U/mL)**				0.866
≤30	14	16.6	7.1–26.3	
>30	49	14.3	9.3–19.3	
**UICC Stage**				**<0.001**
IIa	16	26.9	20.9–21.9	
IIb	53	17.1	12.8–21.5	
III	11	12.5	11.1–13.9	
IV	16	5.1	1.8–8.4	
**Grading**				**0.006**
G1 and G2	46	23.6	15.5–31.6	
G3	34	13.1	8.4–17.9	
**Metastasis**				**<0.001**
M0	80	20.1	16.0–24.2	
M1	16	5.1	1.8–8.5	
**Nodal invasion**				**0.002**
Nx	9	6.0	3.9–8.2	
N0	19	26.4	21.1–31.7	
N1	68	16.7	12.6–20.7	
**Lymphatic invasion**				0.730
L0	39	20.4	14.7–26.0	
L1	31	20.1	12.0–28.2	
**Perineural invasion**				0.061
Pn0	17	29	15.6–42.3	
Pn1	48	19.8	13.3–26.4	
**Vene invasion**				0.800
V0	59	20.1	15.4–24.7	
V1	11	21.6	9.3–33.9	
**Resection margin**				0.521
R0	51	21.6	18.4–24.9	
R1	19	17	11.5–22.4	
**Tumor size (cm)**				0.382
≤3	45	20.4	15.9–24.8	
>3	21	28	13.7-42.2	
**Type of surgery**				**<0.001**
Pancreatic head resection	53	21.8	15.3–28.3	
Pancreatic left resection	9	19.8	11.9–27.8	
Total Pancreatectomy	9	20.1	9.7–30.4	
Excisional biopsy	25	7.9	2.7–13.2	
**Type of chemotherapy**				**<0.001**
Adjuvant	62	21.6	18.1–25.2	
Palliative	25	11.9	10.5–13.4	
No chemotherapy	9	1.9	1.7–2.3	

**Table 2:** Diagnostic performance of single EMT-miRs and CA.19-9 in discriminating PDAC from Non-PDAC. Values are calculated by ROC analysis. *p* ≤ 0.05 indicates significance.

Non-PDAC vs. PDAC				
microRNA	Tissue		Serum	
	AUC	*p*-Value	AUC	*p*-Value
miR-10b	0.656	0.018	0.663	0.010
miR-34a	0.503	0.963	0.640	0.026
miR-141	0.726	<0.001	0.682	0.004
miR-148a	0.885	<0.001	0.534	0.554
miR-197	0.674	0.010	0.627	0.049
miR-200a	0.542	0.434	0.658	0.013
miR-200b	0.723	<0.001	0.792	<0.001
miR-200c	0.838	<0.001	0.780	<0.001
miR-203a	0.550	0.455	0.627	0.047
miR-429	0.537	0.490	0.650	0.019
miR-655	0.646	0.026	0.548	0.452
	**AUC**	***p*-Value**	**Sensitivity**	**Specificity**
**CA.19-9**	0.834	<0.001	0.781	0.870

**Table 3:** Analyzed Epithelial-to-Mesenchymal Transition (EMT)-miRs and their targets.

EMT-regulating microRNA	Target/Function	Ref.
**EMT-Suppressive MicroRNAs**		
miR-34a	Blocks Snail1 and Notch1	[17]
miR-148a	Inhibits the Wnt/β-Catenin pathway	[23]
miR-200-family (-141, -200a, -200b, -200c, -429)	Block ZEB-1 and ZEB-2	[14]
miR-203a	Inhibits the Wnt/β-Catenin pathway	[24]
miR-655	Blocks ZEB-1 and TGF-β-R2	[27]
**EMT-Promoting MicroRNAs**		
miR-10b	Promotes TGF-β-signaling	[28]
miR-197	Blocks p120 catenin (a cooperator of E-cadherin)	[29]

## Supplementary Materials

The following are available online at http://www.mdpi.com/2072-6694/10/9/328/s1. Figure S1: Phenotypes of human PDAC cell lines Mia-PaCa-2 and BxPC-3 by 10-fold magnification in light microscopy. Scale bar: 200 µm; Figure S2: Average gene expression stability measure M for housekeeping genes calculated by geNorm Software; Table S1: Patients of the study.
