# Supplementary material for: Clinical Impact of Epithelial-to-Mesenchymal Transition Regulating MicroRNAs in Pancreatic Ductal Adenocarcinoma
Source: Cancers (Basel). 2018 Sep 13;10(9):328. doi: 10.3390/cancers10090328 (PMC6162771; doi:10.3390/cancers10090328)
Supplement: Supplementary file 1 [file cancers-10-00328-s001.docx]

Supplementary material: Clinical Impact of Epithelial-to-Mesenchymal Transition Regulating MicroRNAs in Pancreatic Ductal Adenocarcinoma

Sameer Abdallah Dhayat, Max Michael Traeger, Jan Rehkaemper, Anda Jana Stroese, Konrad Steinestel, Eva Wardelmann, Iyad Kabar and Norbert Senninger


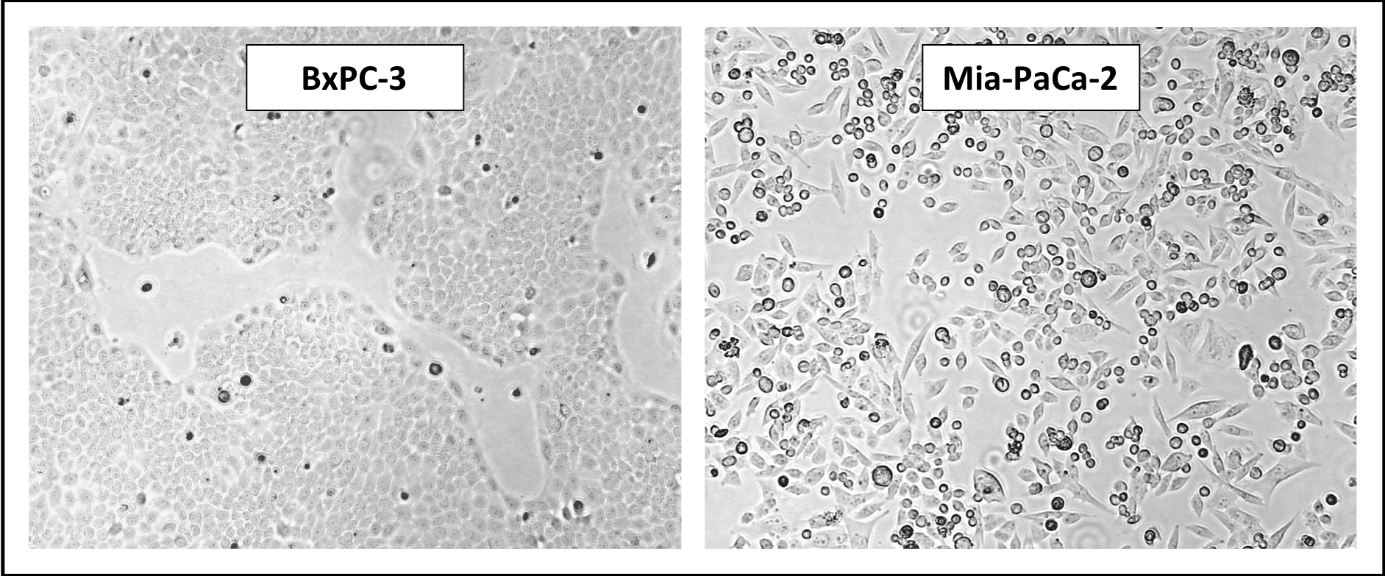


**200µm**

**Figure S1.** Phenotypes of human PDAC cell lines Mia-PaCa-2 and BxPC-3 by 10-fold magnification in light microscopy. Scale bar: 200µm.


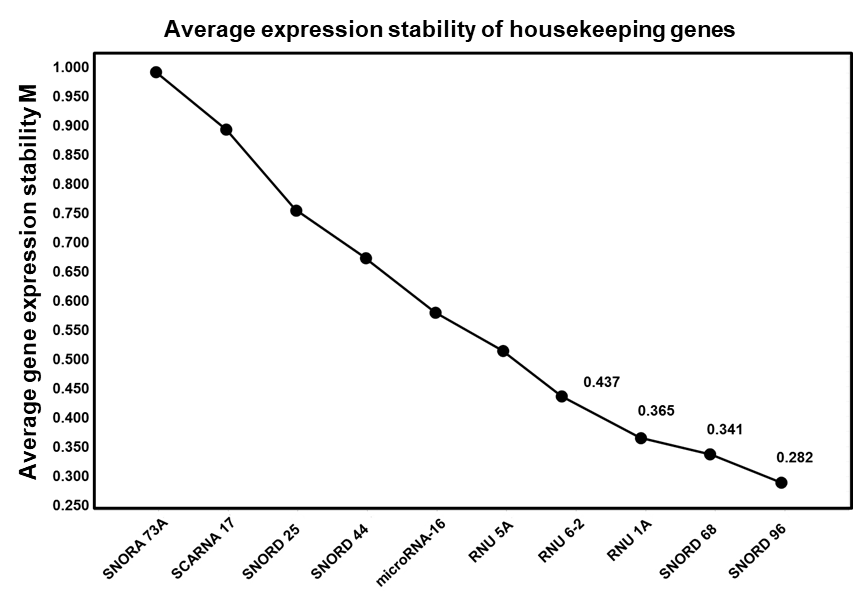


**Figure S2.** Average gene expression stability measure M for housekeeping genes calculated by geNorm Software.

**Table S1. Patients of the study.**

| **Category** | **Total**  **(n=185)** | **Healthy Controls**  **(n=46)** | **Chronic Panreatitis**  **(n=32)** | **IPMN**  **(n=11)** | **PDAC UICC Stage II**  **(n=69)** | **PDAC UICC Stage III**  **(n=11)** | **PDAC UICC**  **Stage IV**  **(n=16)** | **p-value**  **PDAC vs. Non-PDAC** |
| --- | --- | --- | --- | --- | --- | --- | --- | --- |
| **Age (years)** |  |  |  |  |  |  |  | 0.474 |
| Median  (Range) | 64.6  (19.6 – 87.9) | 61.7  (19.6 – 87.9) | 60.4  (34.0 – 80.3) | 74.6  (36.7 – 79.7) | 66.4  (31.1 – 84.8) | 75.0  (49.9 – 82.4) | 69.5  (35.2 – 80.3) |  |
| ≤60 | 73 | 20 | 16 | 2 | 27 | 4 | 4 |  |
| >60 | 112 | 26 | 16 | 9 | 42 | 7 | 12 |  |
| **Gender** |  |  |  |  |  |  |  | 0.133 |
| Female | 86 | 26 | 13 | 8 | 26 | 6 | 7 |  |
| Male | 99 | 20 | 19 | 3 | 43 | 5 | 9 |  |
| **Body Mass Index** |  |  |  |  |  |  |  | 0.235 |
| Median  (Range) | 24.5  (15.9 – 45.8) | 26.2  (17.3 – 36.0) | 23.2  (18.5 – 33.6) | 22.4  (15.9 – 26.8) | 24.1  (18.7 – 37.0) | 24.4  (19.1 – 31.3) | 25.4  (16.7 – 45.8) |  |
| ≤25 | 101 | 16 | 20 | 8 | 42 | 8 | 7 |  |
| >25 | 78 | 26 | 12 | 3 | 25 | 3 | 9 |  |
| **Smoker** |  |  |  |  |  |  |  | 0.333 |
| Yes | 48 | 11 | 13 | 2 | 15 | 3 | 4 |  |
| No | 137 | 35 | 19 | 9 | 54 | 8 | 12 |  |
| **Alcohol Abuse** |  |  |  |  |  |  |  | 0.322 |
| Yes | 13 | 3 | 5 | 0 | 3 | 1 | 1 |  |
| No | 172 | 43 | 27 | 11 | 66 | 10 | 15 |  |
| **Pre-surgical Diabetes mellitus** |  |  |  |  |  |  |  | **0.024** |
| Yes | 47 | 5 | 8 | 3 | 19 | 6 | 6 |  |
| No | 138 | 41 | 24 | 8 | 50 | 5 | 10 |  |
| **Pre-surgical pancreatitis** |  |  |  |  |  |  |  | **<0.001** |
| Yes | 50 | 0 | 32 | 3 | 11 | 2 | 2 |  |
| No | 135 | 46 | 0 | 8 | 58 | 9 | 14 |  |
| **Pre-surgical CA.19-9 (U/ml)** |  |  |  |  |  |  |  | **<0.001** |
| Median  (Range) | 43.3  (0.6 – 18517.4) | 8  (1.6 – 42.9) | 19.4  (7.4 – 3000.0) | 17.7  (13.0 – 23.9) | 201.1  (0.6 – 18517.4) | 176.8  (2.6 – 5245.4) | 1069.4  (20.0 – 17041.2) |  |
| ≤30 | 48 | 13 | 16 | 5 | 10 | 2 | 2 |  |
| >30 | 56 | 2 | 5 | 0 | 31 | 7 | 11 |  |

P-values are calculated by unpaired two-tailed t-test. P ≤ 0.05 indicates significance.
